# Tet-inducible lymphoma in a new Tax transgenic mouse model

**DOI:** 10.1186/1742-4690-8-S1-A4

**Published:** 2011-06-06

**Authors:** Daniel Rauch, Sirosh Bokhari, John Harding, Lee Ratner

**Affiliations:** 1 Division of Molecular Oncology, Washington University in St. Louis, St Louis, MO, 63110, USA

## 

The sufficiency of Tax in cancer initiation is evident in a variety of animal models, but the role Tax plays in cancer maintenance is less well understood. Tax is often undetectable in freshly obtained ATLL cells and while Tax promotes proliferation and protects cells from apoptosis it is also highly immunogenic and cytotoxic. We developed a mouse model in which Tax is expressed in the Tet-On system in vivo. These mice carry 4 transgenes. Tax, under the TET-promoter (TET-TAX) was provided by Warner Greene. The reverse tet activator, coupled to the granzyme B promoter (GZB-rtTA) , drives Tax in activated T and NK cells in a doxycycline (dox)-inducible manner. Two additional transgenes were included for non-invasive imaging. TET-GFP, which is regulated by the same promoter as Tax in this model, serves as a readout for Tax gene expression and firefly luciferase driven by the HTLV-1 LTR (LTR-LUC) serves as a bioluminescent readout for Tax activity. Quadruple transgenic mice were maintained with or without dox-diet for 18 months. Over that time, 6 of 8 animals receiving dox-diet developed marked lymphadenopathy and splenomegaly, compared to 2 of 9 on normal diet. 1 of 9 mice lacking the GZB-rTA transgene and 0 of 7 mice lacking the TET-TAX trangene developed lymphoma. Discontinuation of dox after lymphadenopathy resulted in a decrease in lymph node size and bioluminescence, but remission was transient. This mouse model allows us to regulate and detect Tax activity non-invasively and will enable targeted interrogation of the role of Tax in lymphoma.

